# Brain power comparison between microgravity and head-down tilt bed rest: an electroencephalography approach

**DOI:** 10.1038/s41598-025-26291-8

**Published:** 2025-11-27

**Authors:** María Sevilla-García, Adrián Quivira-Lopesino, Pablo Cuesta, Sandra Pusil, Ricardo Bruña, Patrique Fiedler, Fernando Maestu, Ana Maria Cebolla, Guy Cheron, Katharina Brauns, Alexander C. Stahn, Michael E. Funke

**Affiliations:** 1 Biomedical Imaging Technologies, Universidad Politécnica de Madrid, Madrid, Spain; 2 Center for Cognitive and Computational Neuroscience, Universidad Complutense de Madrid, Madrid, Spain; 3 Department of Pediatrics, McGovern Medical School, The University of Texas Health Science Center at Houston, Houston, TX USA; 4 Department of Experimental Psychology, School of Psychology, Universidad Complutense de Madrid, Madrid, Spain; 5 Department of Radiology, Rehabilitation, and Physiotherapy, School of Medicine, Universidad Complutense de Madrid, Madrid, Spain; 6 Health Research Institute of the Hospital Clínico San Carlos (IdISSC), Madrid, Spain; 7 Institute of Biomedical Engineering and Informatics, Technische Universität Ilmenau, Ilmenau, Germany; 8 Laboratory of Neurophysiology and Movement Biomechanics, Université Libre de Bruxelles, Brussels, Belgium; 9 Charité-Universitätsmedizin Berlin, a Corporate Member of Freie Universität Berlin, Humboldt-Universität zu Berlin, and Berlin Institute of Health, Institute of Physiology, Berlin, Germany

**Keywords:** EEG, Spaceflight, HDBR, Microgravity, Spaceflight analogs, Neuroscience, Neurophysiology

## Abstract

This study examines neurophysiological changes in microgravity by comparing EEG data from two ground analog 60-day head-down tilt bed rest (HDBR) experiments (ESA/DLR “Cocktail” and “RSL”) and the NEUROSPAT experiment in space. The primary objective was to determine whether HDBR could effectively model spaceflight’s impact on the human brain’s EEG signal. In the HDBR dataset, increases in relative delta (2–4 Hz) (*p* < 0.01) and theta (4–8 Hz) (*p* < 0.001) power bands were observed during a two-month HDBR experiment, predominantly in the left temporal and parieto-occipital regions. Conversely, the NEUROSPAT dataset showed a significant increase in beta (12–30 Hz) (*p* < 0.05) power in the left somatosensory cortex during in-flight conditions, suggesting a potential adaptation to disrupted proprioceptive input and motor control in microgravity. The contrasting findings between the two datasets indicate that while HDBR can simulate some aspects of microgravity, it may not serve as a model for all central nervous system changes, especially those related to proprioception and motor functions. This highlights the need for further research, including larger sample sizes, consistent EEG recording conditions, and integration of additional physiological and cognitive markers to fully understand the effects of prolonged microgravity.

## Introduction

Human health and performance during spaceflights are affected by environmental conditions like isolation, radiation, and microgravity^1,2^. These factors not only pose risks to astronaut’s well-being but can also impact mission success. To ensure that crewmembers maintain optimal cognitive and behavioural performance, research must focus on how these conditions impact the central nervous system^3,4^. Developing strategies to mitigate any adverse effects is a priority for exploration missions^5–7^.

Despite an absence of severe neurological impairments during spaceflights missions, astronauts commonly report temporary issues such as motor deficiencies, disorientation, spatial illusions, visual disturbances, reduced performance, and sleep disruptions^8–10^. These subjective experiences seem to align with previously reported brain^11,12^ and electrophysiological changes^13–15^. Additionally, structural MRI studies have shown that astronauts can experience physical brain changes during a six-month mission on the International Space Station (ISS), with estimates suggesting that over 50% of astronauts may be affected^11,12,16^. Therefore, investigating the brain’s functional state throughout space missions may provide key insights into the clinical significance of these effects.

Traditionally, neuropsychological tests have dominated cognitive function assessments in astronauts^3,17,18^. However, they may have limitations in identifying subclinical and preclinical shifts^19^, specially in this high cognitive capacity population. Therefore, monitoring brain function during space flights using techniques like electroencephalography (EEG) is increasingly essential, especially as functional brain changes may precede visible structural alterations^15,20,21^. EEG practical advantages—such as lightweight equipment, the ability for repeated recordings, and high signal quality—make it an ideal tool for directly measuring global neuronal electrical activity. In this context, brain function assessed through classical event-related potentials and EEG power spectral variations related to a specific task has revealed that astronauts onboard the ISS exhibit: (1) stronger alpha EEG rhythms during an eyes-closed resting state^1^, (2) alterations in neural top-down signals during 3D visual perception^2^, (3) decreased alpha rhythms during visual attention in a docking task involving the motor cortex and cerebellum^5^, (4) changes in sleep quality^7^; Project ARIADNA, ESA, 2017–2018), and (5) a decrease in the “P300” brain response, reflecting a reweighting of the emotional valence of success/failure feedback towards neutrality^14^.

EEG offers a reliable, non-invasive method for examining ongoing brain activity in real time, and resting EEG has been successfully used in clinical research^22^. Furthermore, it has already been employed in over 50 previous space missions as part of polysomnography and cognitive studies^23–25^. One of the most common oscillatory characteristics of the brain measured via EEG is power distribution^26^, a quantification of the amount of activity or energy rate in a specific frequency band: delta (2–4 Hz), theta (4–8 Hz), alpha (8–12 Hz), beta (12–30 Hz), and gamma (30–45 Hz).

To better understand how these spaceflight conditions affect the human body and brain, researchers also rely on Earth-based space analogs. Earth-based space analogs are environments or experimental setups that simulate the conditions encountered in space to study their effects on human physiology and performance^6,27^. These analogs, such as head-down tilt bed rest (HDBR)^28–32^, parabolic flights^8,9^, or extreme environments like Antarctica^10^ or deep-sea habitats^33^, are designed to mimic aspects like microgravity, isolation, or confined spaces. By using these analogs, researchers can gather crucial data on how extended space travel might affect astronauts, as well as test strategies for mitigating potential negative impacts. These controlled environments allow the study of both physical and psychological challenges before actual space missions, contributing to the development of effective countermeasures.

One of the most widely used space analogs is HDBR, which specifically simulates the physiological effects of microgravity experienced by astronauts in low Earth orbit (LEO). In space, astronauts experience weightlessness, which causes a significant fluid shift, including cerebrospinal fluid (CSF), towards the head area^34^, along with an increased ventricular volume and an upward movement of the brain^11,16^. HDBR reproduces these effects by positioning participants at a downward angle, with their heads lower than their feet for extended periods, closely mimicking the gravitational changes observed in space. As an experimental model, HDBR is used to study how microgravity impacts various bodily systems, including cardiovascular, musculoskeletal, and central nervous system functions, helping researchers better understand and address the challenges of long-duration space travel^35–37^.

In this work, we present a comparative analysis of EEG data from the two ESA/DLR sponsored 60-day HDBR studies “RSL” and “Cocktail”^28^, and data from astronauts aboard the International Space Station (ISS)^13^, aiming to investigate neurophysiological changes associated with prolonged microgravity and its Earth-based analogs. By exploring the similarities and differences in brain function across these conditions, we aim to deepen our understanding of microgravity’s effects on the central nervous system and assess the validity of HDBR as a reliable analog for studying its impact on human brain activity.

## Methods

In this study, we conducted a retrospective analysis of anonymized data from three distinct datasets: two Head-Down Tilt Bed Rest (HDBR) datasets (Experiment 1 and Experiment 2) and the International Space Station (ISS) dataset.

### Head-down tilt bed rest (HDBR) dataset

The head-down tilt bed rest (HDBR) dataset is a collection of data derived from two distinct experiments conducted within the framework of the DLR research grant awarded to A. Stahn. This meticulously processed dataset can be accessed in the data repository published in^28,29,38^.

The first experiment (Experiment 1) took place at the facility of the German Aerospace Agency (DLR) in Cologne, Germany, during 2015/2016^39^. The goal was to assess the long-term effects of HDBR with and without exercise as a countermeasure. Resting state EEG data were collected from 23 young, healthy, right-handed men. For the current study, 11 participants from the control group, who did not engage in any physical training, were included (demographic data can be found in Table 1). Resting state EEG recordings with eyes closed were taken for 3 min at different time points: seven days before bed rest (BDC-7), on the second day of HDBR (HDBR2), on the 28th day (HDBR28), the 56th day (HDBR56), and 11 days after bed rest condition (*R* + 10) (cp. Table 2).

**Table 1:** Demographic characteristics for both HDBR experimental groups and ISS group at baseline. *Data are presented as means plus/minus standard deviations; N represents the sample size.

	HDBR		ISS
	Experiment 1	Experiment 2	
N	11	10	5
Age	28.3 ± 5.5	33.5 ± 8.3	54.2 ± 2.6
Sex			
Male	11	10	5
Female	0	0	0

**Table 2:** The EEG recording times for each HDBR experiment are shown for three phases: pre-HDBR (second column), HDBR (third column), and post-HDBR (fourth column).

	Pre-HDBR		HDBR		Post-HDBR	
	Experiment 1	Experiment 2	Experiment 1	Experiment 2	Experiment 1	Experiment 2
Time (days)	7	8	2	7	10	7
			28	31		
			56	60		

The second experiment (Experiment 2) was conducted at the French Institute for Space Medicine and Physiology (MEDES) in Toulouse, France, in 2017^40^. The aim was to assess the long-term effects of HDBR with and without antioxidant/anti-inflammatory supplementation as a countermeasure. Resting state EEG data were collected from 20 young, healthy right-handed men. For this study, 10 participants from the control group, who did not receive any supplementation, were included (demographic data can be found in Table 1). Resting state EEG recordings with eyes closed were performed for 3 min at several time points: eight days before bed rest (BDC-8), on the seventh day of HDBR (HDBR7), on the 31st day (HDBR31), the 60th day (HDBR60), and eight days after recovery (*R* + 7) (cp. Table 2).

For this study, we have merged both HDBR datasets (Experiment 1 and Experiment 2) and considered them as a single combined database. This approach allows us to increase the sample size and statistical power. Despite minor differences in the timing of recordings between the two experiments, the EEG acquisition protocols and conditions were sufficiently similar to justify their integration. All analyses were conducted on this unified dataset.

#### Ethics statement

The first bed rest study was registered with the German Clinical Trials Register (DRKS, registration number DRKS00012946, date September 18th, 2017) and received approval from the Ethics Committee of the Northern Rhine Medical Association (Ärztekammer Nordrhein) in Düsseldorf, Germany, as well as the local Ethics Committee at Charité - Universitätsmedizin Berlin, Germany.

The second bed rest study was listed in the ClinicalTrials.gov database (NCT03594799) and was approved by the Comité de Protection des Personnes (CPP Sud-Ouest Outre-Mer I), the French Health Authorities (Agence Française de Sécurité Sanitaire des Produits de Santé), and the Ethics Committee at Charité-Universitätsmedizin Berlin, Germany.

Both studies complied with ethical standards for medical research involving human participants as outlined by the World Medical Association’s Declaration of Helsinki. Prior to participation, all individuals were fully informed about the study objectives, experimental procedures, and possible risks, and provided both verbal and written informed consent.

#### Data acquisition

In both experiments, the brain activity of participants was recorded using electroencephalography (EEG) during a 3-min resting-state session. These measurements took place in dimly lit, soundproof rooms between 8:30 a.m. and 1:30 p.m. Data collection was done with participants seated during baseline and recovery periods and lying in a supine position with a − 6 degree head-down tilt during the head-down bed rest phase. A memory foam cushion was placed under their heads to reduce discomfort from wearing the EEG cap.

EEG data were captured using a 32-channel amplifier (actiCHamp, Brain Products GmbH, Germany) with electrodes attached to an EEG cap (actiCap, Brain Products GmbH, Germany), positioned according to the International 10–10 System^41^. Electrode positions included Fp1, Fp2, F7, F3, Oz, Fz, F4, F8, FT9, FC5, FC1, TP9, CP5, CP1, TP10, CP6, CP2, FT10, FC6, FC2, C3, Cz, C4, T7, T8, P7, P3, Pz, P4, P8, O1, and O2, with Fz used as the reference electrode. Electrode impedance was checked and kept below 5 kΩ before starting data collection.

Eye movements and blinks were tracked using tin electrooculogram (EOG) electrodes (B18 Multitrodes, EASYCAP GmbH, Germany) placed above and below the left eye and at the outer corners of both eyes. EEG and EOG signals were amplified by a multi-channel bio-signal amplifier and digitally sampled at 1000 Hz with 24-bit resolution.

#### Data preprocessing

The EEG signals were processed using a bandpass filter between 0.5 and 65 Hz. To eliminate sinusoidal noise (50 Hz line interference), the CleanLine function^42^ was applied. Recordings were then visually inspected to identify bad channels, which were interpolated using spherical spline interpolation (less than 2% of channels required interpolation). After re-referencing the data to a common average reference, the signals were divided into 4096-ms epochs, with a 10% overlap between consecutive segments. The first and last 5 s of each recording were discarded to avoid potential eye-related artifacts. EOG artifacts were removed through regression of the vertical and horizontal EOG channels^43^. Muscle artifacts were filtered out using a spatial filtering method with default settings^44^. A baseline removal was performed, and an automated rejection process excluded epochs exceeding a gradient threshold of 100 µV or amplitudes outside the range of ± 200 µV. On average, 89% of the epochs were retained for further analysis.

The remaining free of artifacts data were re-segmented into 4-second epochs, with a 2-second overlap. The final dataset contained 40 epochs across all subjects and situations.

### International space station (ISS) dataset

This dataset was derived from the NEUROSPAT study (AO-2004, 118)^9,13,21^, which was conducted between 2011 and 2013. Five right-handed male astronauts (demographic data can be found in Table 1), who spent six months in low Earth orbit (174.6 ± 19.9 days), took part in the study. To ensure similar amounts of sleep prior to the recordings, astronauts completed a sleep questionnaire, and were allotted 8.5 h of sleep the night before the recordings. Data collection was avoided within 48 h of air travel that crossed more than 4 time zones, after work shifts causing a time change greater than 4 h, following sleep deprivation, or after intensive physical or mental activities like centrifuge training, vestibular countermeasures tests, or spacewalks. Astronauts were instructed to maintain their usual caffeine intake and abstain from alcohol or medication for 16 h before the recordings. Each astronaut was asked to perform the recordings at approximately the same time of day, within a ± 2-h window, ideally in the morning. The participants were evaluated at different time points: 67, 43 and 28 days before flight, on the 9th and 55th day in-flight, and 3, 7, 17 and 20 days after returning to Earth (Table 3).

**Table 3:** The average EEG recording times for all five astronauts are shown for three phases: pre-flight (second column), in-flight (third column), and post-flight (fourth column). *Data are presented as means plus/minus standard deviations.

	Pre-flight (on earth)	In-flight (aboard ISS)	Post-flight (on earth)
Time (days)	66.8 ± 9.0	8.8 ± 1.8	3.0 ± 0.4
	42.6 ± 0.9	54.6 ± 3.7	7.0 ± 1.2
	28.0 ± 0.4		16.8 ± 0.6
			20.2 ± 1.0

#### Ethics statement

All experimental procedures were approved by the European Space Agency Medical Care Committee and the NASA Johnson Space Center Institutional Review Board for Human Testing and were conducted in compliance with the Helsinki Declaration. Written informed consent was obtained from all participants before the beginning of the experiment.

#### Data acquisition

The brain activity of all participants was recorded using EEG during a 2-minute task-free eyes closed condition (followed by a 2-min task-free eyes opened condition where gazed was oriented to a cross displayed on a laptop screen)^13^. For the present study, only the eyes-closed recordings were used. On Earth, participants sat comfortably in a chair. In microgravity, they remained in a free-floating position, with a belt around their waist, secured to straps attached to metal rings on both sides of the Columbus module in the ISS, in order to prevent major movements. To eliminate any external visual stimuli, a cylindrical tube connected to the laptop screen and fitted with a face mask was placed over the astronaut’s head. This setup was used for both the ground and space recordings.

EEG data were collected at a sampling rate of 1116 Hz using the 59-channel electroencephalogram mapping module (MEEMM) of the European physiology module, located in the Columbus module of the ISS, at the European Astronaut Center in Köln, Germany, or in Star City, Moscow. The MEEMM employs a dedicated physical reference electrode placed on the right earlobe. For some post-flight recordings at the Johnson Space Center in Houston, a 64-channel asalab amplifier (ANT Neuro BV, Hengelo, Netherlands) was utilized in a standard laboratory setting, with a sampling frequency of 1024 Hz. The asalab amplifier is a stationary DC-EEG device that utilizes a common average reference. Scalp-electrode impedance was measured and maintained below 5 kΩ for all recordings.

#### Data preprocessing

Fifty-five common EEG channels, derived from a subset of the 10–10 system, were selected from both the MEEMM and asalab systems to ensure consistent layout and spatial coverage of the head. Bad channels were automatically detected by analyzing the mean power spectral density (PSD) within the frequency range 70–100 Hz. A channel was classified as bad if its PSD exceeded the mean PSD plus three times the standard deviation of all 55 channels in that recording^45^. Bad channels were then interpolated using spherical splines^46^, and the DC offset for each channel was eliminated. Following the correction of bad channels, the data were re-referenced to a common average reference. Ocular artifacts were identified and removed through principal component analysis (PCA) (ASA software, ANT Neuro BV, Hengelo, Netherlands), targeting components that accounted for 95% of the variance in the noise subspace. Muscle artifacts were automatically detected and eliminated using the FieldTrip package^47^ from Matlab R2023b (version 23.2, https://www.mathworks.com/products/matlab.html ). Additional residual artifacts were discarded through visual inspection by experts. The remaining artifact-free data were divided into 4-second segments with a 2-s overlap. On average, the final dataset contained 23 ± 3 epochs across all subjects and situations. Finally, for the power and functional connectivity analysis, the cleaned EEG time series were filtered within a frequency range of 2 to 45 Hz. This process was performed using a high-order (1500) finite impulse response (FIR) filter with a Hamming window. Additionally, a two-pass filtering approach with 2-second padding was applied to prevent any alterations in the signal characteristics due to filter-induced phase shifts.

### HDBR and ISS datasets

To enable a comparison between the two datasets, the number of channels in the ISS dataset was reduced, selecting only the 32 channels that were common to the HDBR dataset. After preprocessing both datasets, source reconstruction was performed followed by statistical analysis to examine and compare the brain activity patterns in each dataset.

#### Source reconstruction

Source activity was estimated using a template MRI derived from the New York Head (ICBM-NY)^48^, employing a 3-layer boundary element model (BEM) for the head. A regular volumetric grid with a 10 mm spacing source model and standard electrode positions was utilized. The forward model was solved with OpenMEEG^49^. Sources were reconstructed independently for each participant using the exact low-resolution brain electromagnetic tomography (eLORETA) method^50^, applying a regularization factor of 10^− 8^. Each source location was labelled according to the Automated Anatomical Labeling (AAL) atlas^51^. The whole brain anatomical model here used comprised 78 cortical and subcortical areas mapped by the atlas (excluding the cerebellum, basal ganglia, thalamus, and olfactory cortices), resulting in a total of 1202 source positions for subsequent analysis.

The power spectrum for each source was calculated for every trial using the averaged periodogram method with multi-taper based on discrete prolate spheroidal sequences (DPSS) and a smoothing of 1 Hz. For each dataset, relative power was obtained by normalizing the power spectrum at each source location to the total power within the 2–45 Hz range. The average power for the AAL anatomical model in each dataset and each frequency band was calculated by averaging over all epochs and the sources within this anatomical model and corresponding frequency steps. Analyses were performed in the conventional frequency bands, defined to avoid overlap: delta (2–4 Hz), theta (4.25–8 Hz), alpha (8.25–12 Hz), beta (12.25–30 Hz), and gamma (30.25–45 Hz). This resulted in a reconstructed power matrix for each condition, organized by stages x frequency bands x participants.

#### Statistical analysis

Statistical analyses were conducted using Prism 10 software (GraphPad version 10.0.2, https://www.graphpad.com/, San Diego, CA, USA). To obtain a single measurement per condition (pre, experimental condition, and post), the three measurements from the HDBR experiment and the two measurements from the NEUROSPAT experiment were averaged. Similarly, the three pre-flight measurements and four post-flight measurements were averaged to produce single values for each condition. Statistical comparisons were performed separately for each dataset (HDBR and ISS) on the power spectrum values obtained from their respective three conditions. The normal distribution of the data (assessed using the Shapiro-Wilk test) was confirmed, and subsequently a one-way repeated measures ANOVA with Tukey’s multiple comparison test was applied. For repeated measures with reduced sphericity, the Geisser and Greenhouse correction method was employed. Results are presented as mean ± standard deviation (SD), with p values noted as follows: *p < 0.05, **p < 0.01, ***p < 0.001. Additionally, q values with a significance level > 5 corresponds with a minimum *p* = 0.05.

## Results

### HDBR dataset (32 channels)

**Fig. 1:**
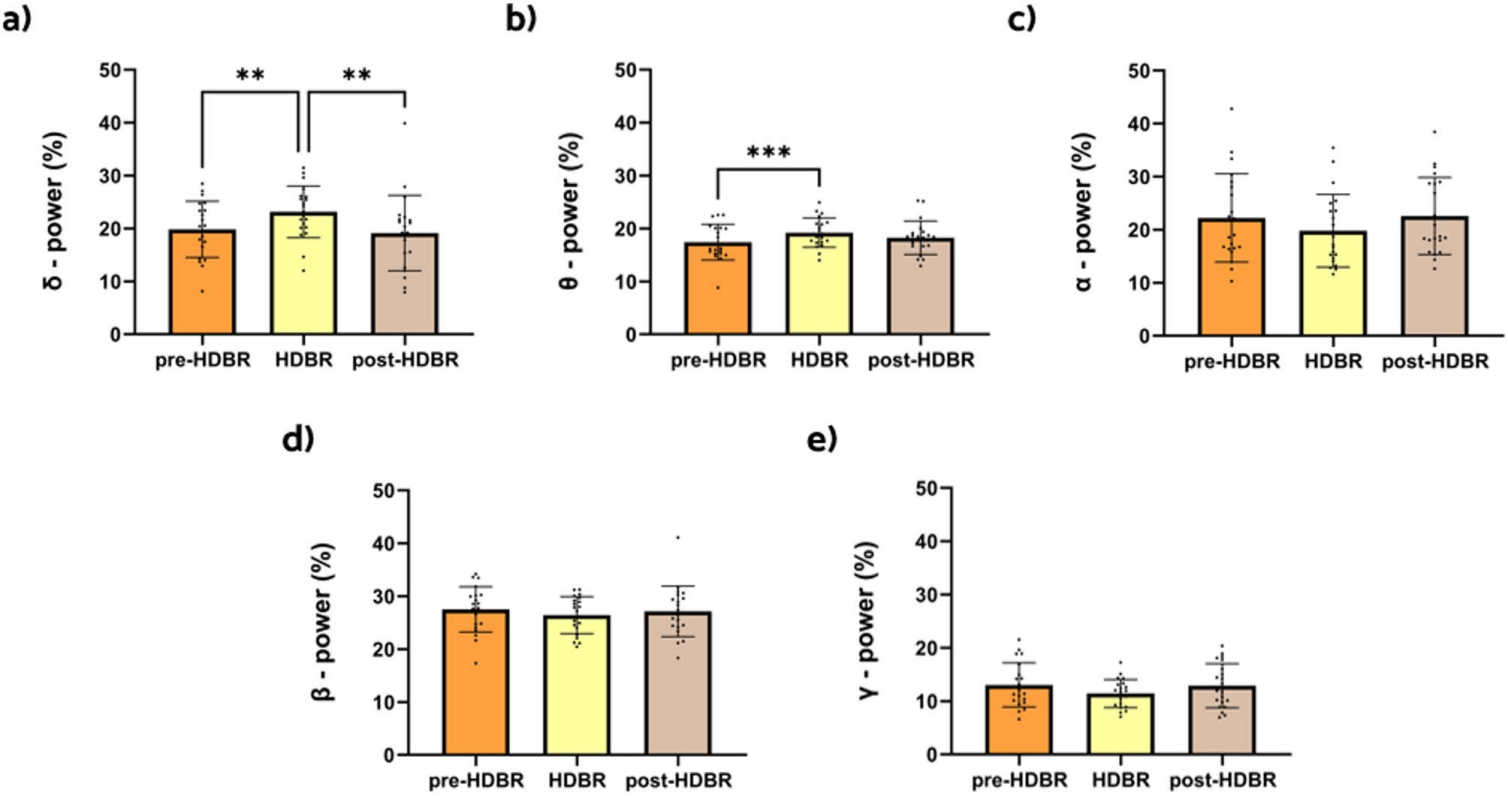
Changes in relative power for all frequency bands between HDBR conditions. The bar graphs depict the mean (in percentage) ± SD of the corresponding band power for each condition (*p < 0.05, **p < 0.01, ***p < 0.001). (**a**) Statistical comparison between conditions for the delta band (2–4 Hz). (**b**) Statistical comparison between conditions for the theta band (4.25–8 Hz). (**c**) Statistical comparison between conditions for the alpha band (8.25–12 Hz). (**d**) Statistical comparison between conditions for the beta band (12.25–30 Hz). (**e**) Statistical comparison between conditions for the gamma band (30.25–45 Hz).

When analyzing the results in the HDBR dataset, the only frequency bands that showed statistically significant results were delta and theta (Figs. 1a,b). Relative delta power was found to be significantly increased during the HDBR condition when compared to the pre-HDBR (*p* < 0.01) and post-HDBR conditions (*p* < 0.01). Relative theta power only showed an increase in the HDBR condition when compared to the pre-HDBR condition (*p* < 0.001). Even though alpha, beta, and gamma did not yield significant results, they appear to show a slight decrease during the HDBR condition (Fig. 1c–e).

**Fig. 2:**
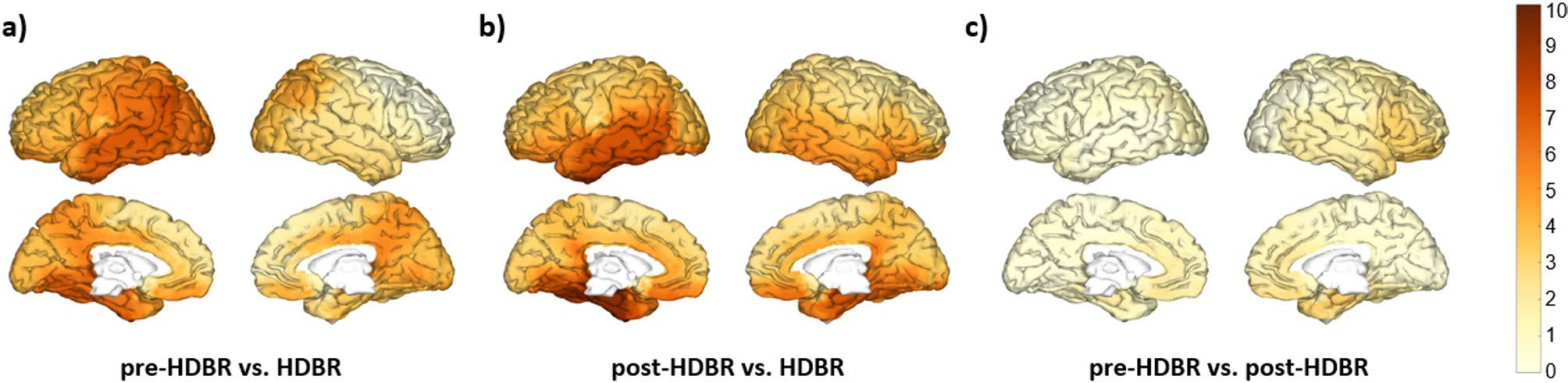
Changes in delta band relative power between conditions. Brain figures represent the areas with higher statistical power changes in the delta band when comparing (**a**) pre-HDBR versus HDBR conditions, (**b**) post-HDBR versus HDBR conditions, (**c**) pre-HDBR versus post-HDBR conditions. The colorbar is displayed as a family-wise corrected significance level of q value > 3.5, corresponding with a minimum *p* value of 0.05. The q statistic value was obtained from the results of the post-hoc Tuckey test of the multiple comparison corrections. Thus, the darker the orange color, the higher the statistical significance of the brain regions.

Regarding delta band, the left temporal cortex and parieto-occipital regions, including the angular gyrus, showed the most considerable increases in power when comparing the HDBR and pre-HDBR conditions (Fig. 2a). For the comparison between HDBR and post-HDBR, the most remarkable increments were found in the left temporal cortex and the bilateral hippocampus and parahippocampus (Fig. 2b).

**Fig. 3:**
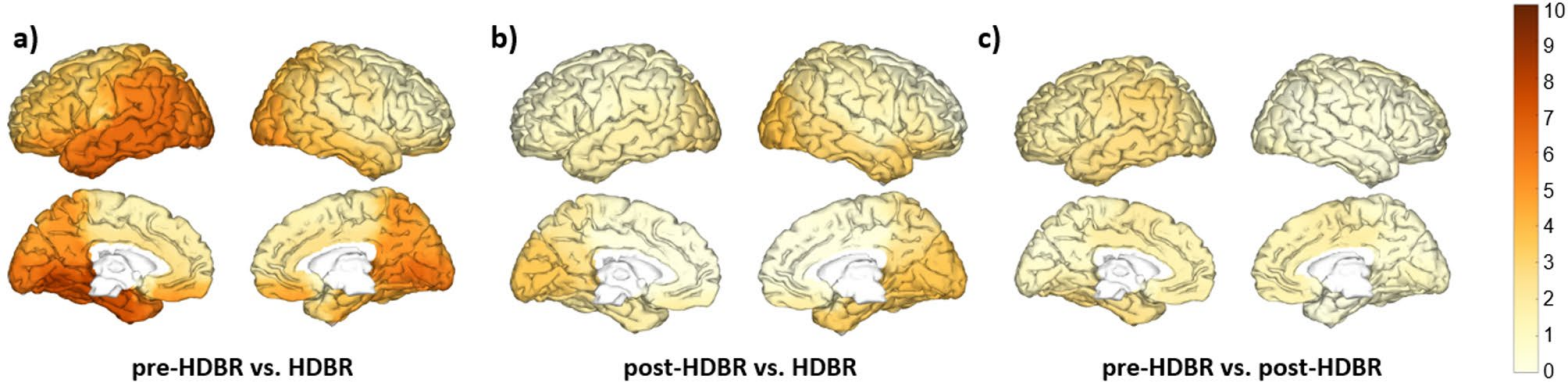
Changes in theta band relative power between conditions. Brain figures represent the areas with higher statistical power changes in the theta band when comparing (**a**) pre-HDBR versus HDBR conditions, (**b**) post-HDBR versus HDBR conditions, (**c**) pre-HDBR versus post-HDBR conditions. The colorbar is displayed as a family-wise corrected significance level of q value > 3.5, corresponding with a minimum *p* value of 0.05. The q statistic value was obtained from the results of the post-hoc Tuckey test of the multiple comparison corrections. Thus, the darker the orange color, the higher the statistical significance of the brain regions.

When examining the theta band, the left temporal cortex and parieto-occipital regions showed the most considerable increases in theta band power when comparing the HDBR and pre-HDBR conditions (Fig. 3a).

### ISS dataset (32 channels)

**Fig. 4:**
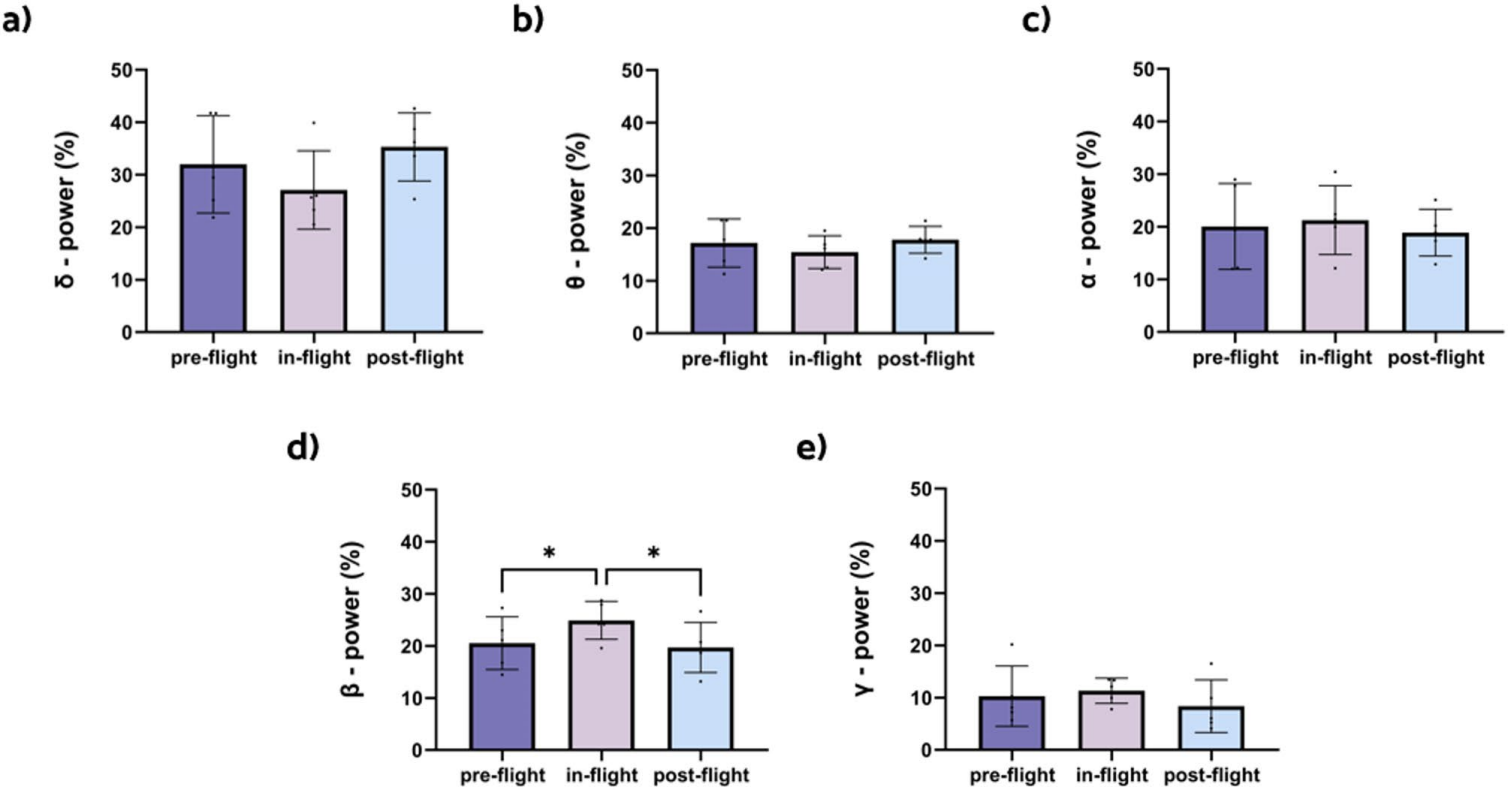
Changes in relative power for all frequency bands between flight conditions, calculated with 32 channels. The bar graphs depict the mean (in percentage) ± SD of the corresponding band power for each flight condition (*p < 0.05, **p < 0.01, ***p < 0.001). Results of the (**a**) statistical comparison between conditions for the delta band (2–4 Hz). (**b**) Statistical comparison between conditions for the theta band (4.25–8 Hz), (**c**) statistical comparison between conditions for the alpha band (8.25–12 Hz), (**d**) statistical comparison between conditions for the beta band (12.25–30 Hz), (**e**) statistical comparison between conditions for the gamma band (30.25–45 Hz).

When analyzing the ISS dataset with 32 channels, the only frequency band that showed statistically significant results was beta (Fig. 4d). Relative beta power was found to be significantly increased during the in-flight condition when compared to both the pre-flight (*p* < 0.05) and post-flight conditions (*p* < 0.05). Even though delta, theta, alpha, and gamma did not show significant results, delta and theta appear to have a slight decrease during the in-flight condition (Figs. 4a,b), while alpha and gamma appear to have a small increase during the in-flight condition (Fig. 4c,e).

**Fig. 5:**
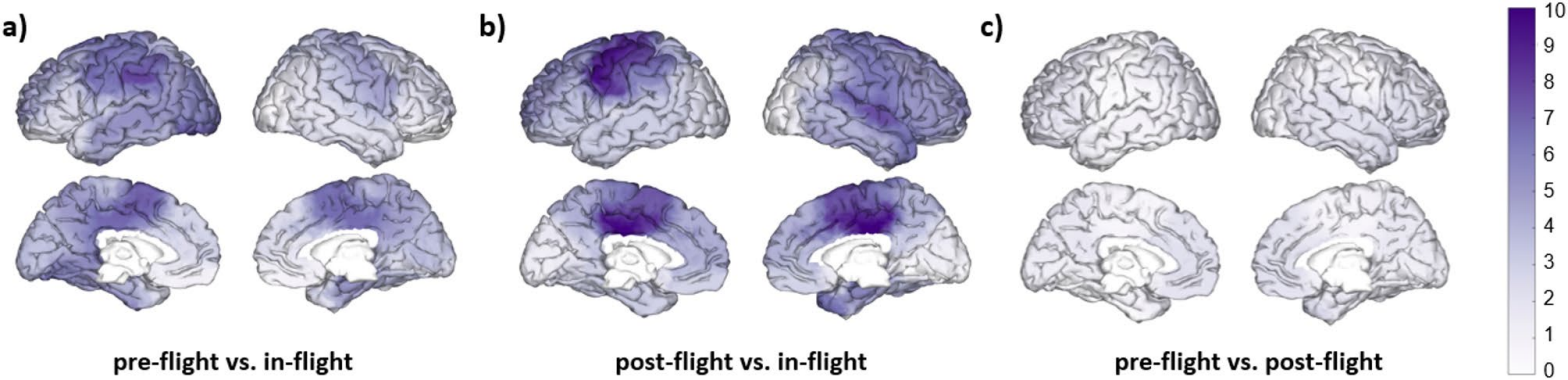
Changes in beta band relative power between flight conditions, calculated with 32 channels. Brain figures represent the areas with higher statistical power changes in the beta band comparing (**a**) pre-flight versus in-flight conditions, (**b**) post-flight versus in-flight conditions, (**c**) pre-flight versus post-flight conditions. The colorbar is displayed as a family-wise corrected significance level of q value > 5, corresponding with a minimum *p* value of 0.05. The q statistic value was obtained from the results of the post-hoc Tuckey test of the multiple comparison corrections. Thus, the darker the blue color, the higher the statistical significance of the brain regions.

Left motor and parietal regions showed the most considerable increases in beta band power when comparing the in-flight and pre-flight conditions (Fig. 5a). For the comparison between in-flight and post-flight, the most remarkable increases are found in the left somatosensory cortex, and the middle part of the cingulate gyrus on both sides (Fig. 5b).

## Discussion

The main objective of this study was to examine the resting state brain activity in two distinct electroencephalography (EEG) datasets: one collected during two prolonged head-down tilt bed rest (HDBR) experiments, i.e., the “RSL” and “Cocktail” studies, and the other from the NEUROSPAT experiment conducted in microgravity. The aim was to identify statistically significant differences in resting brain activity before and after the experimental conditions. Additionally, the study sought to compare these datasets to evaluate whether HDBR could serve as an effective Earth analog for studying the effects of microgravity on human brain physiology.

Recently, Brauns et al.^28^, found significant reductions in absolute power during HDBR across delta, theta, alpha, and beta frequency bands that occurred as early as after 24 h of bed rest, and remained decreased during HDBR. The authors attributed these changes to a brain shift and redistribution of cerebrospinal fluid^11^. In contrast, the present study analysed relative power of electrocortical activity, i.e., the proportion of activity within a specific frequency band compared to the total power across all combined bands, in the same HDBR dataset.

Our findings showed increases in the relative power of delta and theta frequency bands during bed rest (Fig. 1a,b). Delta waves, typically associated with deep sleep and rest, reflect a slowed state of brain activity^52^, while theta waves, linked to relaxation and memory, indicate reduced cognitive demand^52–54^. Notably, these changes predominantly manifested in the left temporal and parieto-occipital regions of the brain (Figs. 2 and 3). The temporal region’s involvement, which is crucial for memory and language, and the parieto-occipital regions, responsible for sensory and visual integration, suggest that these areas experience increased theta and delta activity during inactivity^52,54^. The increased delta and theta power in the left temporal and parieto-occipital regions during HDBR suggest a reduction in cognitive and sensory processing, with implications for memory and sensory integration over time^52,54^. The increase observed in the present study does not contradict the original findings but are attributed to different representations of spectral power, i.e., total power in the original work vs. relative power in the present paper.

However, some studies have shown that changes in body position, such as moving from a supine to an upright posture, can reduce low-frequency EEG components, including delta and theta waves^55,56^. This indicates that postural differences during data collection could influence the results. In our study, the pre- and post-sessions were conducted while participants were seated, whereas the HDBR recordings were performed at a − 6° head-down tilt, which may have contributed to the observed effects. Further investigation is needed to fully understand how these position-related differences impact brain activity patterns during prolonged bed rest.

In contrast to the results from the HDBR study, the ISS dataset—where channels were reduced to 32 to match the bed rest study—showed a significant increase in beta band power during in-flight conditions (Fig. 4d). This increase was primarily localized in the left somatosensory cortex (SMC) (Fig. 5). Beta frequency oscillations are known to play a key role in motor control and proprioception^57,58^. In addition, the SMC is known to play a role in proprioceptive processing and motor function and learning^59–62^. In microgravity, the proprioceptive sensory inputs, such as vision, touch and vestibular signals^63^, are significantly disrupted^5^. It has been observed that the otolith’s function in the vestibular system, which relies on gravity, is altered in the absence of gravitational cues, while somatosensory inputs, like touch, are diminished unless astronauts hold onto or stabilize themselves^64^. These disruptions force astronauts to adjust by relying more on visual feedback^11,65^, prompting functional adaptations in the brain. The increased beta band power observed in the left SMC during flight might indicate that these proprioceptive changes may affect brain activity function, further reinforcing the idea that beta oscillations are integral to adapting motor control in microgravity environments^66^.

Because beta activity has been implicated in both maintaining posture and suppressing movement^67^, the observed effects might also result from differences in postural tone across conditions. During the pre- and post-sessions participants were seated, while in space they remained afloat, restrained only by a waist belt. Fully clarifying whether these postural differences account for the results will require additional investigation.

These disparities in results suggest that, in certain scenarios, HDBR may not fully replicate the changes induced by microgravity, particularly in the central nervous system (CNS)^67^. Our findings indicate that a -6° HDBR condition cannot be considered a direct analog of actual microgravity for studying CNS adaptations, as the results do not align perfectly. However, this discrepancy is observed in this specific case, highlighting the need for additional data to confirm or reject this conclusion. To develop more reliable procedures, it would be ideal to conduct pre- and post-HDBR recordings under standardized − 6° tilt conditions across experiments. This approach would allow for more robust within-HDBR comparisons across time points and also enable direct comparison of pre- and post-conditions between experiments. By establishing consistent experimental principles and endpoints, and assuming the HDBR results are valid under proper experimental conditions, this framework would help determine whether genuine differences exist between the prolonged HDBR condition and actual spaceflight. Furthermore, addressing such inconsistencies would benefit from standardizing equipment, procedures, and analysis pipelines across studies to ensure the comparability and reliability of results.

This study leaves many questions unanswered due to several limitations. The two in-flight recordings were taken only during the first two months of a six-month space mission, making it difficult to determine when changes occur and how they evolve throughout the entire flight. Additional recordings during the remaining four months would have been valuable to understand the progression of microgravity effects over time. The duration of these changes, as well as the time needed to revert to baseline, also remains unclear since the follow-up period ended just 20 days post-return to Earth. Furthermore, the ISS dataset included only a small number of participants, lacking gender diversity as no female participants were included—despite evidence that neurophysiological data can vary by gender^69,70^. Another limitation lies in the difficulty of directly comparing EEG results from HDBR and spaceflight, as findings suggest dissimilarities between these environments; thus, the physiological effects of simulated microgravity on Earth may not fully capture those in actual space conditions. Compounding these challenges is the lack of cognitive, structural, cardiovascular, or physiological markers that might help clarify these findings. For a more complete understanding of the onset and persistence of these changes, future research should include larger and more diverse cohorts that may include MRI scans before and after the flight, blood sample analyses pre- and post-flight, cortisol measurements, inclusion of both sexes, a higher number of recordings taken before, during, and after the flight, and an overall larger sample size. In addition, we suggest the establishment of a minimum standard EEG protocol for resting-state recordings in spaceflight studies, which would ensure greater consistency and comparability across future investigations. Implementing these measures would greatly enhance our understanding of the physiological and neurocognitive effects of microgravity.

## Data Availability

The HDBR datasets analysed in this study are available in online repositories. The repository name(s) and accession number(s) can be accessed at: 10.6084/m9.figshare.12148359. The NEUROSPAT experiment dataset can be requested from the Laboratory of Neurophysiology and Laboratory of Neurophysiology and Movement Biomechanics at Université Libre de Bruxelles, subject to approval from the European Space Agency Medical Board (ESA-MB) and the NASA Johnson Space Center Institutional Review Board (NASA-IRB). The authors do not own the data; however, interested researchers may request access through ESA and NASA. The authors confirm that they had no special access or privileges beyond those available to others.
